# The Impact of Coping Style on Gaze Duration

**DOI:** 10.1371/journal.pone.0015395

**Published:** 2010-11-15

**Authors:** Tim Klucken, Anne-Marie Brouwer, Astros Chatziastros, Sabine Kagerer, Petra Netter, Juergen Hennig

**Affiliations:** 1 Bender Institute of Neuroimaging, University of Giessen, Giessen, Germany; 2 TNO Human Factors, Soesterberg, The Netherlands; 3 Max-Planck-Institute for Biological Cybernetics, Tuebingen, Germany; 4 Department of Psychology, University of Giessen, Giessen, Germany; Kyushu University, Japan

## Abstract

The understanding of individual differences in response to threat (e.g., attentional bias) is important to better understand the development of anxiety disorders. Previous studies revealed only a small attentional bias in high-anxious (HA) subjects. One explanation for this finding may be the assumption that all HA-subjects show a constant attentional bias. Current models distinguish HA-subjects depending on their level of tolerance for uncertainty and for arousal. These models assume that only HA-subjects with intolerance for uncertainty but tolerance for arousal (“sensitizers”) show an attentional bias, compared to HA-subjects with intolerance for uncertainty and intolerance for arousal (“fluctuating subjects”). Further, it is assumed that repressors (defined as intolerance for arousal but tolerance for uncertainty) would react with avoidance behavior when confronted with threatening stimuli. The present study investigated the influence of coping styles on attentional bias. After an extensive recruiting phase, 36 subjects were classified into three groups (sensitizers, fluctuating, and repressors). All subjects were exposed to presentations of happy and threatening faces, while recording gaze durations with an eye-tracker. The results showed that only sensitizer showed an attentional bias: they gazed longer at the threatening face rather than at the happy face during the first 500 ms. The results support the findings of the relationship between anxiety and attention and extend these by showing variations according to coping styles. The differentiation of subjects according to a multifaceted coping style allows a better prediction of the attentional bias and contributes to an insight into the complex interplay of personality, coping, and behavior.

## Introduction

The processing of negative stimuli is associated with the vulnerability to psychological disturbances. For instance, Davidson showed that patients suffering from depression cannot inhibit negative associations and spend more time perceiving and remembering information with threatening or self-esteem reducing content, which can also be measured by different psycho-physiological parameters [1]. Accordingly, some studies reported that certain coping styles and emotion regulation techniques (e.g. suppression) [2]–[4] could be a potential risk factor for mood and anxiety disorders [5]–[7]. Variations in attentional bias (often defined as reaction time towards threat-inducing stimuli minus reaction time towards positive or neutral stimuli) is one of the most studied processes for a better understanding for fast emotional processing in anxiety disorders [8]–[11].

Recent meta-analyses [12], [13] on studies investigating attentional bias towards threat reported a moderate or even no effect of anxiety trait on attentional bias. One important reason for this small or lack of effect might be the faulty assumption that high-anxiety (HA) subjects show a consistent attentional bias, whereas low-anxiety (LA) subjects do not react with an increased attention to threat. In contrast to this assumption, current anxiety models, like Krohne's repressor-sensitizer model, assume that different copings styles (and not trait anxiety per se) influence the response towards threatening stimuli [14], [15]. Thus, this model explicitly allows different hypotheses for the response patterns of HA-subjects by including a multifaceted coping style (e.g. [14], [15]; for a review on similar current models see: [16], [17]).

According to Krohne's Model, two orthogonal, global reaction systems determine individual coping and behavior style: vigilance and arousal intolerance. These can, for instance, be measured by the Mainz Coping Inventory (MCI; [15]). Vigilance coping behavior can be characterized by a set of behavior strategies reducing uncertainty triggered by a high degree of ambiguity inherent in threatening situations [14], [15]. The second global reaction system is defined as intolerance for arousal. Subjects intolerant to arousal try to avoid distressing stimuli inducing emotional arousal. Thus, intolerance for arousal could be characterized by the avoidance of threatening stimuli, whereas vigilance reactions comprise seeking and monitoring such cues [14]. According to Krohne's model, it is important to consider the combination of both scales (vigilance and arousal intolerance) in order to make assumptions about behavior. Four possible coping styles arise when the vigilance and the cognitive avoidance dimensions are combined:

(1) “Sensitizers” (intolerance for uncertainty but tolerance for arousal) manifest consistent vigilant behavior and direct their attention continuously to the threat relevant information. (2) “Repressors” (tolerance for uncertainty but intolerance for arousal) are characterized by avoiding such threatening cues. The remaining two styles, (3) “low-anxiety subjects” (tolerance for uncertainty and tolerance for arousal) and (4) “fluctuating subjects” (intolerance for uncertainty and intolerance for arousal) are characterized by very fluctuating (or flexible) behavior, which leads to less predictive behavior. For instance, it is assumed that fluctuating subjects switch very fast from approaching to avoiding behavior and vice versa due to arousal but also uncertainty intolerance [14], [15]. However, these last two coping styles have only barely been investigated so far [15], [18].

Previous studies frequently investigated the relationship of high trait anxiety and attentional bias with typical anxiety trait questionnaires, which are mostly based on vigilance items ([19]–[23]; for overviews see: [12], [24]). The problem is that these questionnaires do not measure individual coping styles so that subjects cannot be classified accordingly (i.e. sensitizers, repressors, fluctuating subjects). Specifically, sensitizer and fluctuating subjects would show nearly the same scores in the anxiety questionnaires, as well as repressors and low anxiety subjects [14], [15], [18], [25]. According to Krohne's model, it is important to distinguish between these four groups. Consequently, measuring tolerance for uncertainty and arousal might be important for getting specific hypotheses in response to aversive stimuli.

The aim of the present study was to explore the influence of coping behavior on gaze duration combining the scales tolerance for uncertainty and tolerance for arousal. At first, we selected subjects with clear coping styles: sensitizers, repressors and fluctuating subjects. Thus, this study included one non-anxious group (repressors) and two anxious groups (fluctuating and sensitizers), where the latter two groups differ with respect to their tolerance for arousal. We tested for significant differences in gaze duration by presenting threatening and happy faces simultaneously. Faces were controlled for arousal so that potential effects would be only based on the difference in valence. It was hypothesized that only sensitizers, but not the other two groups, would show approaching behavior reflected in longer gaze duration for threatening faces. Most of the previous studies analyzed the attentional bias in the first 500 ms. Yet, since current studies have started to investigate longer exposure times (cf. [12], [26]), two time windows were of interest: The first 500 ms after stimulus onset were defined as the first time window; the second time window – defined as the remaining time before stimulus offset - was of explorative interest.

## Results

### 500ms

**Figure 1:**
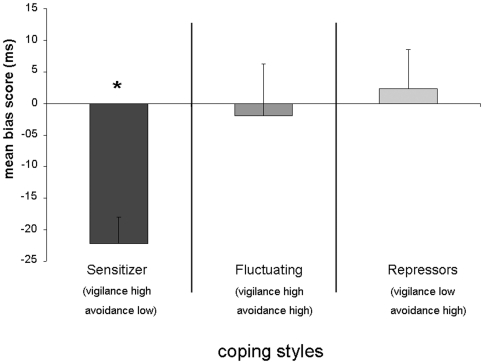
Mean bias scores (gaze duration viewing positive faces – gaze duration viewing threatening faces) of sensitizers, fluctuating subjects, and repressors (and standard errors of the mean) during the first interval 500 ms. The asterisk indicates that the bias score significant differs from zero. **: *p*<.01.

Regarding the first 500 ms, the analysis of variance yielded significant effects of coping style indicating longer gaze durations to the threatening than to the happy faces in the sensitizer group (*F*_(2,35)_ = 4.11; *p*<.05; see figure 1). Post-hoc analyses revealed a significant difference between sensitizers and repressors (*p* = .01) and between sensitizers and fluctuating subjects (*p*<.05). However, correcting for multiple comparisons, the comparison between sensitizers and fluctuating subjects can only be regarded as a trend. Further, one sample t-tests showed that only sensitizers showed a bias towards the threatening faces (*t*_(11)_  = 5.23; *p*<.001; see figure 1), whereas as repressors and fluctuating subjects did not show an significant bias score (both *p*>.70).

### 500–8000 ms

**Figure 2:**
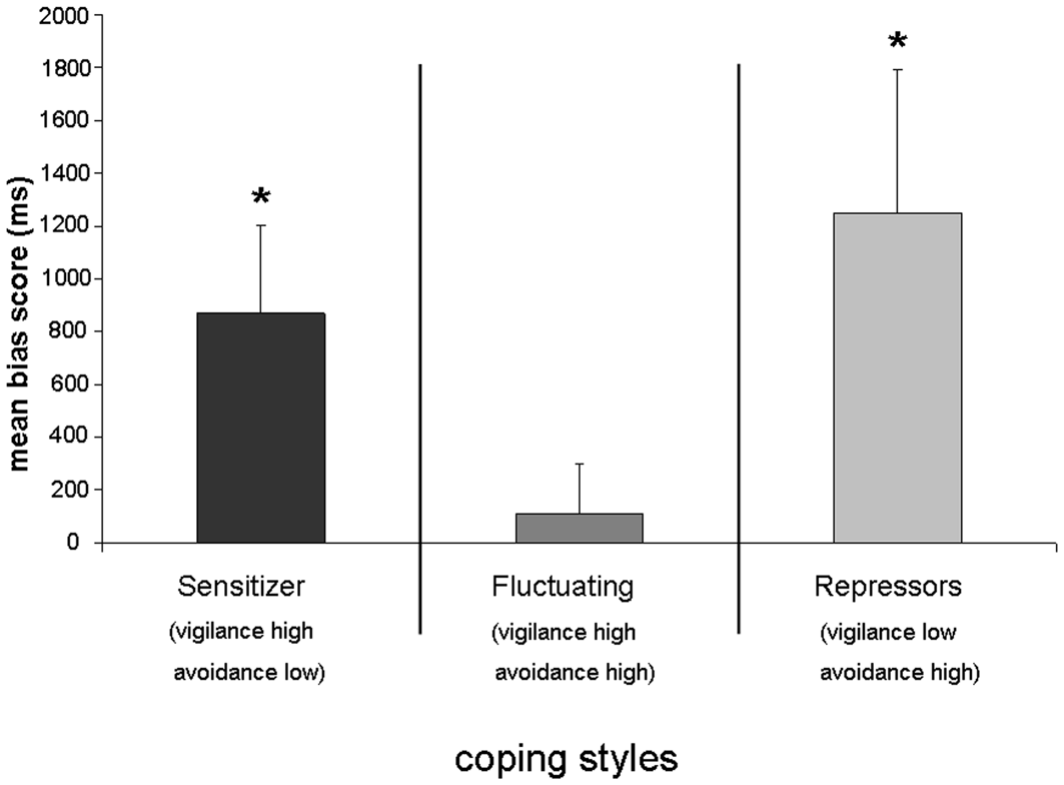
Mean bias scores (gaze duration viewing positive faces – gaze duration viewing threatening faces) of sensitizers, fluctuating subjects, and repressors (and standard errors of the mean) during the interval 500–8000 ms. The asterisks indicate that the bias scores significant differ from zero. **: *p*<.01. *: *p*<.05.

This interval was analysed to investigate whether the significant differences observed in the first period would still be valid when analyzing the time interval after 500 ms. We did not find a main effect of coping style (*F*_(2,35)_  = 2.26 ; *p*>.05), although a trend could be observed, indicating that fluctuating subjects might differ from repressors. One sample *t*-tests revealed that repressors and sensitizers showed a bias towards positive faces (both *p*<.05; figure 2) whereas fluctuating subjects did not show any bias at all (*t*_(11)_  = 0.62; *p*>.50).

## Discussion

The influence of coping styles on various aspects of human functions such as memory [27]–[29], responses of the autonomic nervous system [30], and even neural activity [31] has gained attention. Previous behavior studies have reported only a small impact of anxiety on attentional bias or even failed to find any influences altogether ([20], [21]; meta-analysis: [12]). We hypothesized that one reason for this finding is that these studies failed to take coping style into account. We explicitly tested the assumption that only subjects with a specific consistent anxiety coping style (sensitizers) but not fluctuating subjects would show an attentional bias. The results revealed that indeed, only sensitizers' gaze was initially drawn towards threatening faces whereas the gaze of the other HA-group (fluctuating subjects) or repressors was not. Interestingly, we found longer gaze durations towards positive faces only in repressors and sensitizers for a window later in time (500–8000 ms), when conscious control processes presumably play a more important role.

The results support the idea that coping style and not exclusively anxiety (relating to the vigilance scale in the MCI, which is high in sensitizers and fluctuating subjects) impacts gaze duration. It extends previous findings of the importance of anxiety for attentional bias by differentiating different forms of high-anxiety traits according to coping style.

Several studies have reported effects of self-described anxiety on attentional bias and gaze duration [7], [26], [32]–[34]. In addition, investigating intolerance for uncertainty and arousal, Ioannou et al. [6] reported a significant bias towards threat in sensitizers, but not in repressors, further supporting our results. Moreover, in a well conducted study, Calvo and Avero [26] reported an early attentional attraction to negative pictures in high anxiety subjects as compared with low anxiety subjects. We extended their findings by showing that only sensitizers react with approaching behavior to aversive stimuli.

Our findings highlight the influence of different coping styles in early perceptual processes [16], [17]. This supports Krohne's model [14] suggesting that the measurement of both coping styles (uncertainty and arousal tolerance) allows for a better prediction of individual behavior than the measurement of trait anxiety by one scale only. Our results are in line with evidence supporting this model by emphasizing an important role of coping styles in the early steps of information processing [17], [18]. The initial attraction of attention towards threatening faces in sensitizers may be explained by the sensitizers' intolerance for uncertainty while they can deal with the arousal this causes. The extensive monitoring of threatening stimuli may allow sensitizers to react earlier and more intensely towards threatening stimuli. For the initial 500 ms, it might be difficult to control one's eye movement by conscious processes. This orienting reaction probably occurs automatically and unconsciously. Thus, subjects may perceive “something somehow threatening” before they really “see” a stimulus.

Currently, there is evidence for the influence of sensitizers' and repressors' coping styles on behavior when confronted with aversive stimuli [17]. Summarizing these results, Derakshan and colleagues [17] postulated a two-level account (“vigilance-avoidance theory”) indicating a vigilant reaction in repressors and a following late avoidance response to threat. For instance, in a series of well-controlled memory experiments, Johnson et al. [28] reported a “paradoxical rebound effect of coping styles” on memory tests in a large sample. In their study, repressors showed retrieval advantages for negative slides in contrast to sensitizers or control groups, which seems to contradict our results. However, we suggest that Johnsons' findings and the present results extend the understanding of coping style on behavior: Current studies have repeatedly reported differences in early engagement and disengagement to salient stimuli, followed by disengagement to these stimuli [12], [35]. Consequently, one could suggest that individual coping style impacts the different aspect of behavior (e.g., eye movements vs. memory) differently. The aim of the study by Johnson et al [28] was not to investigate such early perceptual processes; thus, variations in attentional bias might more reflect preattentive responses to threat (e.g. within the first 500ms of stimulus presentation), whereas memory is based on different (longer-lasting) processes like consolidation, retrieval and recall. Therefore, one could suggest that the first eye movements are more affected by the primary coping styles and only after that a rebound effect occurs for prolonged responses like memory processes.

As a limitation, only 36 participants with a robust coping style could be identified, resulting in a rather small sample size (12 participants in each group). This is just above the minimally suggested group size for the used statistical tests. Hence, studies with a larger sample size are needed to investigate the present findings in more detail. Further, we only used one negative facial expression (threatening faces). However, sensitizers might also show prolonged gaze duration towards other negatively stimuli like disgust-inducing faces. In addition, an alternative explanation for the present findings might be that sensitizers “want” to avoid positive pictures and do not prefer negative pictures (as we hypothesized), resulting in the same results. A comparison between negative pictures with neutral pictures (and/or positive pictures with neutral pictures) could clarify this point. It should also be kept in mind that the present study investigated non-clinically high-anxious subjects, not a clinical sample (e.g. generalized anxiety disorders). Hence, it is not entirely clear whether the present findings can be generalized to a clinical sample. It is still being investigated whether response patterns in clinical and non-clinical samples are comparable (cf. [12], [36]).

In sum, we found an influence of coping style on gaze duration in early perceptual processes. We made an effort to control our stimuli for arousal such that the effects are caused by valence and not arousal differences. We would like to point out that the combination of the different established tests (e.g. memory tests) with novel paradigms and methods [33], [37] like measuring gaze duration contributes to a better understanding of the mechanisms underlying individual responses to threat [33]. The results indicate that recording eye movements is a suitable method to investigate coping behavior, allowing more insight into the complex interplay of coping, perception, and behavior.

## Materials and Methods

### Participants

Each recruited subject signed a written informed consent informing them that they could terminate the experiment at any time. None of the subjects had a history of psychiatric or neurological disorders. The study was in accordance to the Declaration of Helsinki. This project was approved by the institutional review board of the University of Giessen and each participant received 10 Euros for participation.

The MCI is based on a categorical approach. In a pretest phase, subjects (*n* = 128) were classified according to their MCI scores, on the basis of the standardized and validated scores of the MCI handbook In order to include only subjects with a distinct coping style, repressors were only classified as such if they scored above 65% on the “cognitive avoidance” scale and below 35% on the “vigilance” scale. Sensitizers scored in the opposite direction (less than 35% on “cognitive avoidance” and 65% or more on “vigilance”). Fluctuating participants scored above 65% on both scales. Participants with other possible scores were excluded. The selection procedure resulted in groups of 12 consistent repressors, 12 consistent sensitizers, and 12 fluctuating subjects (each group included 6 males and 6 females). All subjects were recruited from the database of an experimental study at the Max Planck Institute Tuebingen. The total sample (*n* = 36) had a mean age of 26.75 years (*SD* = 5.12). The mean age did not differ significantly between the three groups (*p* = .48). Each subject had normal or corrected to normal vision.

In addition to the MCI, the recruited participants filled in the Multidimensional Personality Questionnaire, which was part of an undergraduate thesis (MPQ; [38], [39]; for a brief version see: [40]). Although the aim of the present study was the impact of coping style on emotional processing as measured by gaze duration, we also determined scores on the stress reaction scale for the different groups since this scale is related to anxiety behavior [38]–[40]. Sensitizers and fluctuating subjects scored significantly higher on the stress reaction scale compared to repressors (*p*<.01). There was no difference between sensitizers and fluctuating subjects (p>.50).

### The Mainz Coping Inventory (MCI)

The MCI is a self-report questionnaire assessing the frequency of using vigilant and cognitive avoiding strategies in several different situations. Subjects have to judge which coping strategies they prefer in a particular situation on a dichotomous true-false scale. Answers are added up resulting in a vigilance (i.e. approaching behavior) and a cognitive avoidance score. Validation, psychometric properties, and factor structures have repeatedly been confirmed [15].

### Stimuli

The Max-Planck video face database is a highly standardized database consisting of 1000 photographs of different kinds of emotional faces [41]. This database provides the possibility to use different emotions displayed by the same person. The pictures had previously been rated for arousal and valence by 15 different experts. A set of 180 (90 happy and 90 threatening) emotional faces of comparable arousal levels were chosen.

### Procedure

Each trial started with a fixation point, which stayed for 1000 ms in the middle of the screen. This was followed by the presentation of one happy and one threatening face with identical arousal values side by side for 8000 ms. Gaze duration at the happy and the threatening face was recorded for each trial. In each of the 90 trials, both faces were presented at the same distance to the fixation point (1.5 cm) on a 36.5×27.5 cm screen. Each stimulus was shown only once. The same number of happy and threatening faces was presented on the left and on the right side of the screen. The computer generated a random order of pairs of faces for each participant. A pseudo randomized stimulus order was used with the restriction that no more than three presentations of the same emotion occurred on the same screen side and both emotions were equally distributed on each side within 30 trials.

After signing the informed consent form, participants filled out the MCI. They were then informed about the next part of the experimental procedure as follows: “In the following experiment, we will present a fixation point on the computer screen. Please concentrate carefully on it. As soon as the point disappears, two faces will appear on the screen. You will not be required to do anything specific, just look at the faces as you like. First, we will have a short training session in order to explain the procedure. If you have any questions, you may turn to the experimenter”. After the briefing, a short training procedure with 3 trials (not including any pictures from the main experiment) was performed.

### Measurement of gaze duration

To record gaze duration, we used a remote, video-based eye tracker (iViewX RED, SensoMotoric Instruments Inc.), which illuminated the eye with infrared light and monitored the corneal reflection of the eye via a video camera. The typical spatial gaze position accuracy is in the range of 0.5°–1.0°. Eye movements were tracked with a sampling rate of 50 Hz. The eye-tracking system was placed in front of the monitor, which was again placed in front of the observer at a distance of 0.5 m without obscuring parts of the image. A calibration procedure was performed for every participant. Gaze positions were stored on a hard disk after each experimental trial for offline analysis.

The dependent variable was defined as the individual bias score, which is the total gaze duration spent on threatening faces minus the gaze duration spent on happy faces. Similar variables were used in previous studies [6], [12], [42]. Trials with data missing for more than 5 s were discarded in order to avoid unwanted influences (e.g. closed eyes). Less than 8% of the trials were discarded. In some studies, others parameters as gaze duration were measured (e.g. first fixation probability, cf. [26]). We chose gaze duration for several reasons. First, we wanted to investigate an early attentional shift (within the first 500 ms) using a variable that would also be suitable to measure a prolonged preference shift (over the last 7500 ms). For this, gaze duration seemed to be a better variable than fixation probability. This is in line with Calvo and Avero [26] showing that different kind of eye movement parameters correspond.

We performed two analyses of variance with coping style as group factor (sensitizer/repressor/fluctuating) and bias score as dependent variable: one for the first time period (the initial 500 ms) and one for the second time period (500–8000 ms). Appropriate post-hoc group comparisons, corrected results for multiple comparisons, were carried out as well. Further one-sample *t*-tests were conducted to test if the bias score significantly differed from zero. All post-hoc tests were performed separately for the first 500 ms (early interval) and the remaining 7500 ms (late interval).
